# High Grade (Large Cell) Neuroendocrine Carcinoma of the Nasopharynx: Novel Case Report with Touch Preparation Cytology and Positive EBV Encoded Early RNA

**DOI:** 10.1155/2015/231070

**Published:** 2015-05-10

**Authors:** Charles D. Sturgis, Brian B. Burkey, Suhael Momin, Aaron P. Hoschar

**Affiliations:** ^1^Cleveland Clinic, Tomsich Pathology Institute, 9500 Euclid Avenue L25, Cleveland, OH 44195, USA; ^2^Cleveland Clinic, Head and Neck Institute, 9500 Euclid Avenue A71, Cleveland, OH 44195, USA

## Abstract

Fewer than five case reports of primary large cell neuroendocrine carcinoma of the nasopharynx are known to the authors. No previous reports have included examples of cytomorphology or have proven association with Epstein-Barr virus. We herein illustrate MRI findings, histopathologic features, immunohistochemical characterization, cytologic details, and in situ hybridization studies from a unique case of primary large cell neuroendocrine carcinoma of the nasopharynx in a 38-year-old Caucasian male patient. Recognition of rare tumor types of the nasopharynx allows for refinements in disease management and prognostication.

## 1. Introduction

The current World Health Organization (WHO) classification of tumors of the head and neck lists three categories of primary malignant epithelial neoplasms in the nasopharynx. These categories are nasopharyngeal carcinomas (NPC), nasopharyngeal papillary adenocarcinomas, and salivary gland-type carcinomas [1]. The first category of nasopharyngeal carcinoma is subdivided into “nonkeratinizing” carcinoma, keratinizing squamous carcinoma, and basaloid squamous carcinoma. The “nonkeratinizing” group is generally further divided into differentiated and undifferentiated (more common) subtypes [1]. In examples of older literature, nasopharyngeal carcinomas are sometimes referred to as lymphoepithelial carcinomas or undifferentiated carcinomas with lymphoid stroma. Common symptoms and physical signs of NPC include neck masses (enlarged lymph nodes), nasal obstruction with bleeding, and sometimes aural changes. A near constant association exists between NPC and Epstein-Barr virus (EBV) infection with strong evidence to support a carcinogenic role of the virus. The presence of host incorporated EBV can be of diagnostic importance and also has therapeutic implications [1, 2]. While they do exist, neuroendocrine carcinomas of the nasopharynx are not included in the WHO classification. Such tumors are known to exist but are seen rarely and can mimic other blue cell tumors such as lymphomas, neuroblastomas, mucosal melanomas, and primitive neuroectodermal tumors [3]. In the lungs (where neuroendocrine tumors are more common), these neoplasms can be divided into carcinoid tumor, atypical carcinoid tumor, large cell neuroendocrine carcinoma, and small cell carcinoma. Case reports of nasopharyngeal primary tumors ranging the morphologic spectrum of these neuroendocrine lung tumors have been reported [4–7]. To our knowledge, only three cases of primary large cell (non-small cell) neuroendocrine carcinoma of the nasopharynx have been reported in the literature, and the reports of these have not included examples of cytomorphology (as opposed to histomorphology) or substantiated associations with EBV [8, 9]. We herein report an EBV positive case of primary large cell neuroendocrine carcinoma of the nasopharynx in a 38-year-old man in which touch preparation cytology studies were integral to establishing a definitive diagnosis.

## 2. Case Presentation

**Figure 1:**
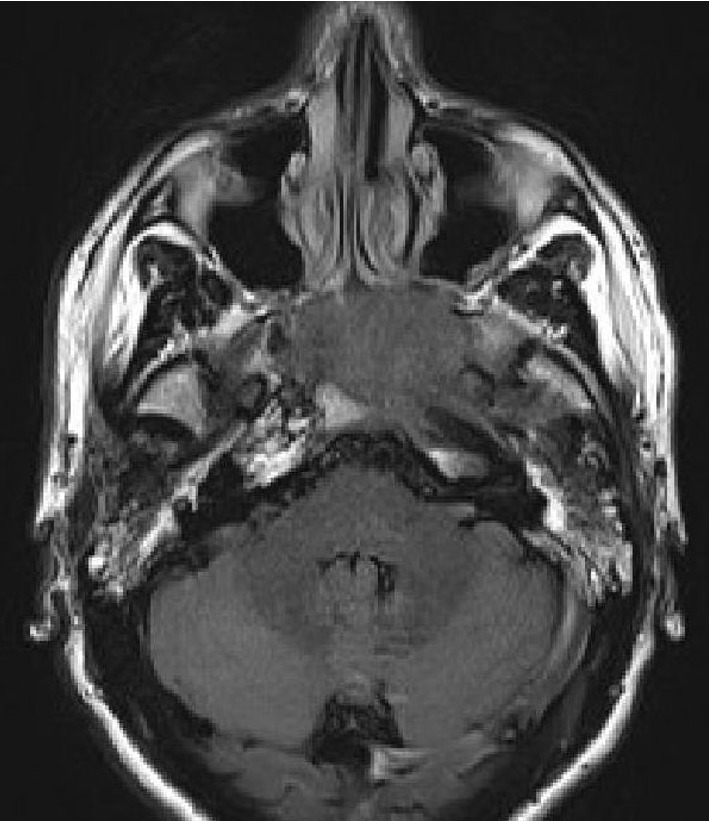
Magnetic resonance imaging of the head and neck revealed a nasopharyngeal mass with extension into the left pterygopalatine fossa. Bony involvement of the greater wings of the sphenoid and clivus was described.

The patient is a 38-year-old, single, Caucasian male who presents with a 2-month history of painless bilateral neck swelling and “muffled” hearing. He reports being recently previously treated with a course of oral antibiotics without resolution of symptoms. He is a current, every-day cigarette smoker with a 15-pack-year history of tobacco use. The patient denies weight loss, fever, night sweats, fatigue, head ache, and visual disturbances. Firm submandibular masses (5 to 8 cm, bilaterally) are reported on physical examination. Imaging studies of the head and neck demonstrate bulky homogenous soft tissue mass-like lymphadenopathy in the bilateral neck as well as a nasopharyngeal mass with erosive changes of the left pterygopalatine fossa and left skull base. Imaging studies are interpreted as “concerning for lymphoma” (Figure 1). Endoscopic nasopharyngeal biopsies are performed.

## 3. Discussion

**Figure 2:**
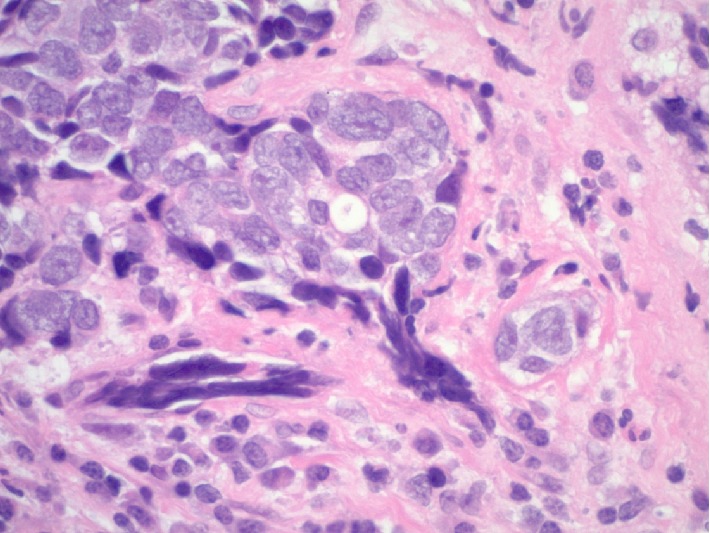
Tissue section from the nasopharyngeal biopsy showed a nested histoarchitecture with crush artifact and a well-preserved region of lesional cells set in a fibrous stroma with accompanying lymphocytes and plasmacytoid cells. Tumor cell sizes were intermediate to large with minimal to moderate amounts of amphophilic cytoplasm, large nuclei with finely granular chromatin, and eosinophilic nucleoli (hematoxylin and eosin stain, 600x).

**Figure 3:**
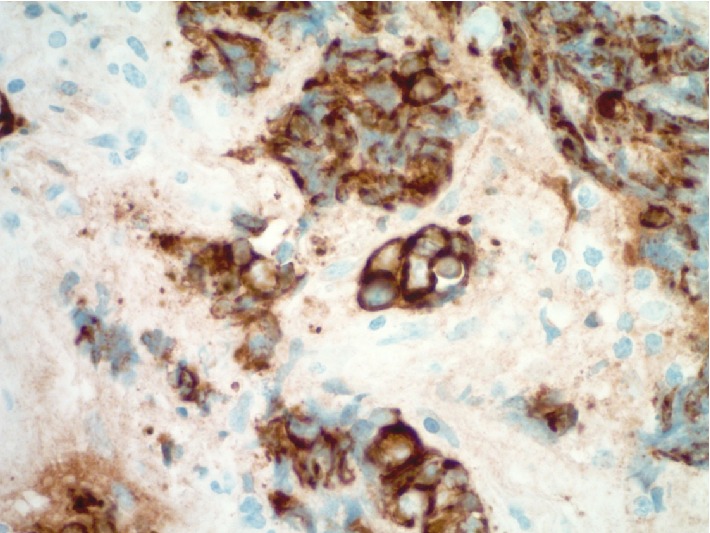
Ancillary testing revealed strong and diffuse tumor cell immunoreactivity for cytokeratin (CAM5.2 immunohistochemistry, 600x).

**Figure 4:**
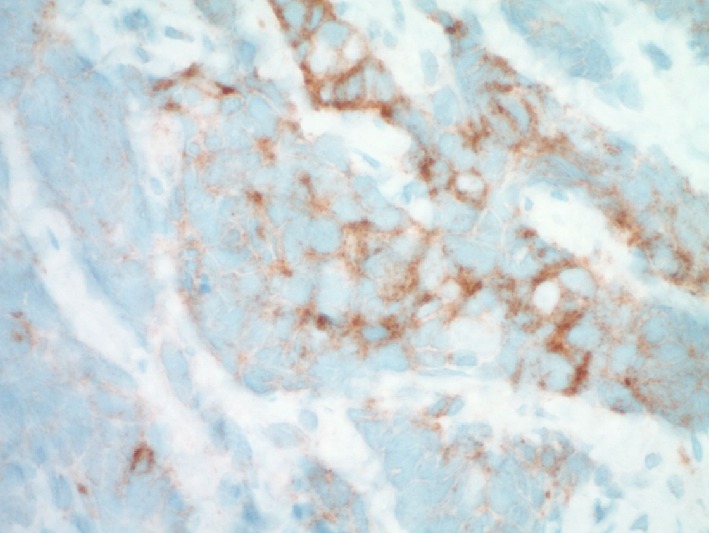
Ancillary testing confirmed immunoreactivity with synaptophysin (synaptophysin immunohistochemistry, 600x).

**Figure 5:**
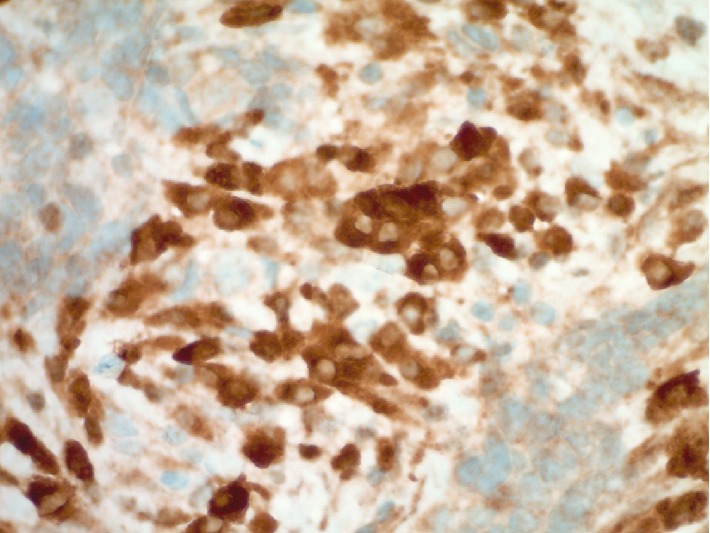
Ancillary testing demonstrated strong immunoreactivity with neuron specific enolase (NSE immunohistochemistry, 600x).

Multiple biopsies were obtained from a nasopharyngeal mass in this case. The tissue sample measured 2.0 × 1.0 × 0.4 cm in aggregate. The specimen was sent fresh to the frozen section area for intraoperative consultation, and both touch preparations and frozen sections were performed on portions of the sample, confirming the presence of lesional material. The neoplastic proliferation was felt to be nonlymphoid at the time of intraoperative assessment, and the entire sample was then submitted for routine histologic processing. Hematoxylin and eosin stained tissue sections from the permanent tissue block revealed marked crush artifact with more than 80% of tumor nests being significantly distorted. In focal areas, well-preserved sheets of lesional cells were seen set within a fibrous/desmoplastic stroma that contained mixed lymphoid cells. The lesional cells were intermediate to large in size and showed a variably solid to nested histoarchitecture with foci of necrosis. Rare vaguely acinar structures were noted. The lesional cells were noted to have fine, uniform chromatin which when coupled with the appearance of the crush artifact suggested a possible neuroendocrine phenotype (Figure 2). Mitotic counts were somewhat difficult because of crush artifact. Results of counts confirmed eight mitoses on average per 10 high power (40x objective) microscopic fields. Ancillary immunohistochemical (IHC) studies were performed, including cytokeratin AE1/AE3, cytokeratin CAM 5.2, cytokeratin 5/6, CD34, CD99, CD45, CD56, neuron specific enolase, synaptophysin, chromogranin, p63, S-100, desmin, and myogenin. The lesional cells (in both the well-preserved and crushed regions) showed diffuse and strong immunoreactivity for cytokeratin CAM5.2, supporting an epithelial phenotype (Figure 3). Cytokeratin AE1/AE3 was also positive. In addition, the lesional cells were shown to be immunoreactive with the pan-neuroendocrine markers neuron specific enolase, synaptophysin, and CD56 (Figures 4 and 5). All other IHC marker studies were nonreactive. Ki-67 studies were not pursued. The combined histomorphology and pattern of immunoreactivity were consistent with a high grade neuroendocrine carcinoma. With the limitations caused by crush artifact and the morphology of the cellular process, the two chief differential diagnostic considerations were small cell carcinoma and large cell neuroendocrine carcinoma.

**Figure 6:**
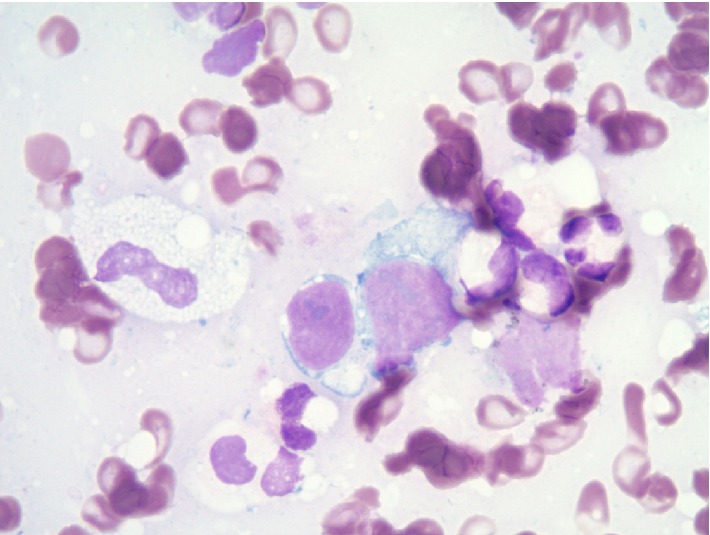
Large cell neuroendocrine carcinoma cytomorphology with moderately abundant pale blue to amphophilic cytoplasm, intermediate to large nuclei, and readily identified nucleoli (Modified Giemsa Stain, Touch Preparation, 1000x).

**Figure 7:**
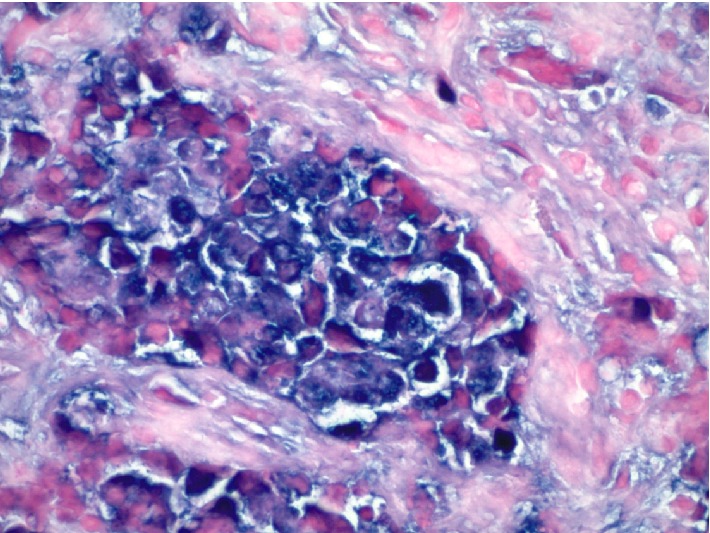
Positive chromogenic in situ hybridization for Epstein-Barr virus encoded early RNAs (slide based EBER CISH study, 600x).

The WHO classification of tumors of the lungs indicates that large cell neuroendocrine carcinomas show histologic features such as organoid nesting, trabecular growth, and palisaded patterns with the tumor cells generally being large in size and having moderate to abundant cytoplasm with nucleoli being present [10]. Characteristic cellular features of large cell neuroendocrine carcinoma of the lungs have also been described in needle aspiration and imprint cytology studies with emphases placed on nuclear size, nuclear/cytoplasmic ratios, and perceptibility of nucleoli in tumor cells [11–13]. Touch preparations were made during the time of the intraoperative consultation in this case. In some instances, touch preparations are used to establish the presence of lesional material and do not later influence diagnostic algorithms. In this case, the touch preparations were an important diagnostic adjunct, and preserved cells in the touch preparations were judged to be of larger size and greater cytoplasmic volume that would be seen in classical small cell carcinoma as well. In addition, lesional cells were noted to contain readily perceived nucleoli. These findings were even more evident on the cytology preparations than in the traditional histologic biopsies. To our knowledge, this report is the first in the literature specifically depicting the cytologic characteristics of cells from a primary nasopharyngeal large cell neuroendocrine carcinoma (Figure 6). In addition to cytologic, histologic, and immunohistochemical characterizations, the tumor in the case was also characterized as being EBV associated by confirming the presence of EBV encoded early RNAs (EBERs) (Figure 7). To our knowledge, this report is the first in the literature confirming an EBV related primary large cell neuroendocrine carcinoma of the nasopharynx.

While most commonly encountered in the lungs, primary high grade neuroendocrine carcinomas can occur at other body sites, including the salivary glands and other sites in the head and neck [14, 15]. High grade neuroendocrine tumors of the head and neck include small cell carcinomas and large cell neuroendocrine carcinomas. Some investigators have reported that, stage for stage, patients with large cell neuroendocrine cancers of the lung survive longer than patients with small cell cancers [16]. Separating high grade neuroendocrine tumors into small cell and non-small cell categories may have diagnostic and prognostic importance. Accurate diagnosis of these rare lesions requires careful clinicopathologic correlations as well as incorporation of pertinent radiographic and historic data. Metastases from a neuroendocrine primary at a distant site should be excluded. Light microscopic interpretations can be effectively enhanced by cytohistologic comparison of touch preparation slides, and ancillary studies including immunohistochemistry and chromogenic in situ hybridization studies for EBER are important in tumor typing.
